# A null mutation in *CABP4* causes Leber’s congenital amaurosis-like phenotype

**Published:** 2010-02-10

**Authors:** Mohammed A. Aldahmesh, Mohammed Al-Owain, Faisal Alqahtani, Salwa Hazzaa, Fowzan S. Alkuraya

**Affiliations:** 1 Department of Genetics, King Faisal Specialist Hospital and Research Center, Riyadh, Saudi Arabia; 2 Department of Medical Genetics, King Faisal Specialist Hospital and Research Center, Riyadh, Saudi Arabia; 3 Department of Surgery, King Faisal Specialist Hospital and Research Center, Riyadh, Saudi Arabia; 4 Department of Pediatrics, King Khalid University Hospital and College of Medicine, King Saud University, Riyadh, Saudi Arabia; 5 Department of Anatomy and Cell Biology, College of Medicine, Alfaisal University, Riyadh, Saudi Arabia

## Abstract

**Purpose:**

To describe the finding of a novel calcium binding protein 4 (*CABP4*) mutation in a family with Leber congenital amaurosis (LCA) phenotype.

**Methods:**

Homozygosity mapping was performed in a consanguineous family with four affected members originally referred as cases of LCA. Detailed electroretinographic recordings were obtained.

**Results:**

A novel homozygous single base-pair insertion was identified in all four siblings. The patients had an LCA-like phenotype, including either flat or greatly diminished electroretinographic activity.

**Conclusions:**

This report significantly expands on the phenotype associated with calcium binding protein 4 mutations, which has so far been limited to congenital stationary night blindness, and further demonstrates how molecular data often blur the boundaries between what are believed to be clinically distinct retinal disorders.

## Introduction

The remarkable organization of retinal cells in layers ensures efficient transmission of the phototransduction cascade that is initiated in photoreceptors to second- and third-order neurons, before it is transmitted through the optic nerve. Perturbation of the phototransduction cascade or its transmission adversely affects vision. The resulting phenotype is known as congenital stationary night blindness (CSNB). Unlike retinal dystrophies, CSNB represents a functional retinal defect, since the clinical appearance of the retina in these patients is largely normal and a detailed functional assessment of the retina is required to make the diagnosis. Several proteins involved in phototransduction (TRPM1 [OMIM 603576], RHO [OMIM 180380], GNAT1 [OMIM 139330], PDE6B [OMIM 163500], GRK1 [OMIM 180381], and SAG [OMIM 181031]) have been implicated in the pathogenesis of CSNB [1-6]. Similarly, mutations in proteins involved in the communication between photoreceptors and the surrounding neurons are also etiologic (CACNA1F [OMIM 300110], NYX [OMIM 300278], and GRM6 [OMIM 604096]) [7-9]. These proteins are encoded by genes that are both autosomal and X-linked, which explains why CSNB follows different modes of inheritance. Encoding calcium-binding protein 4 (*CABP4*) was recently identified by the candidate gene approach as a disease gene in autosomal recessive CSNB [10]. Several characteristics made this an attractive candidate: 1) its cellular localization to the synaptic terminals of photoreceptors [11]; 2) its physical association with and capacity to modulate the activity of Cav1.4α, a calcium channel that mediates the synaptic release of the neurotransmitter glutamate from photoreceptors in the dark [11]; and 3) the CSNB phenotype in *Cabp4* knockout mice [12]. Compared to the wild type, *Cabp4^−/−^* mice manifest a 50% reduction of the “a” wave (generated by photoreceptors), and their “b” wave (generated by bipolar cells) was even more markedly reduced [12]. So far, a total of three human mutations have been reported in this gene [10,13]. Despite the variability in the phenotype associated with these mutations, electroretinography (ERG) findings were consistent with CSNB. The purpose of this paper is to describe a novel frameshift mutation in *CABP4* predicted to be more severe in nature, compared to those reported previously. The four patients had a clinical presentation highly reminiscent of Leber Congenital Amaurosis (LCA, congenital onset, nystagmus, severe visual impairment, and severely diminished or extinguished ERG), which expands the spectrum of the *CABP4*-associated phenotype.

## Methods

### Human subjects

Four siblings (3 sisters and 1 brother) ranging in age from 6 to 16 years were referred to King Faisal Specialist Hospital and Research Center for workup of nystagmus, photophobia, and decreased visual acuity; the referral diagnosis was LCA. They were recruited under an IRB-approved research protocol (REC#2070023, KFSHRC) and a written informed consent was signed by each of the subjects. All four patients underwent a comprehensive ophthalmological evaluation that included best-corrected visual acuity (VA), slit-lamp examination, funduscopy, and full-field ERG (in accordance with International Society for Clinical Electrophysiology of Vision standards) under both scotopic and photopic conditions. Dilated fundoscopic photographs were acquired using Topcon Fundus Camera (Topcon Medical Systems Inc., Paramus, NJ).

### DNA extraction and genotyping

DNA was extracted from whole blood using a Gentra Puregene Blood Kit (Qiagen, Valencia, CA). Genomewide genotypes were obtained using the Affymetrix SNP 250K Chip platform (Affymetrix, Santa Clara, CA) following the manufacturer’s instructions. Blocks of homozygosity were identified using the Affymetrix® Genotyping Console™ (Affymetrix) as described in previous studies [14,15]. Briefly, we use the default settings of the Genotyping Console™ to identify runs of homozygosity. These are cross checked against a comprehensive list obtained from the literature of all genes known to cause hereditary retinal disorders to prioritize genes for sequencing.

### Mutation analysis

*CABP4* was PCR-amplified using primers that covered the entire coding sequence, as well as the flanking intronic sequences. PCR amplification was performed on a thermocycler (Applied Biosystems, Foster City, CA) in a total volume of 25 µl. PCR primers, as well as reaction conditions, are available upon request. PCR amplicons were submitted for bidirectional sequencing using an Amersham ET Dye Terminator Cycle Sequencing Kit (Amersham Biosciences; Piscataway, NJ) following the manufacturer’s instructions. Sequence analysis was performed using the SeqManII module of the Lasergene (DNA Star Inc., Madison, WI) software package, with a normal sequence used for comparison.

## Results

### Clinical phenotype

**Figure 1:**
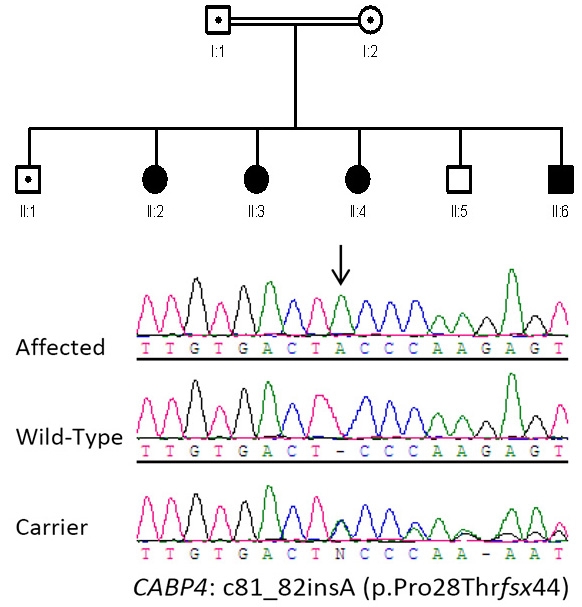
Family pedigree with a novel *CABP4* mutation. The upper panel shows the pedigree of a consanguineous family with four affected children. The square denotes male, the circle denotes female, the central dot indicates the carrier state, and the solid filling indicates the affected state. The lower panel shows sequence chromatogram with the site of insertion indicated by the arrow. Carrier and Normal (same family) are shown for comparison.

Four siblings from a single Bedouin family were enrolled in this study, ranging in age from 6 to 16 years. The parents are first cousins and belong to a large tribe in the central region of Saudi Arabia. To the best of our knowledge, there is no history of CSNB in the extended family members. The parents were unaffected and the sibship includes two unaffected siblings (Figure 1).

The index patient (II-3) was a 15-year-old Saudi girl with an unremarkable perinatal history. The family noticed her poor vision around the age of 2–3 months, evidenced by nystagmus and lack of fixation and tracking. She continued to have very poor vision as a child and, despite prior evaluations, no specific diagnosis was given to this patient except that she had a “retinal disorder.” There was a history of photophobia, but not night blindness; color vision was normal. Our opthalmological evaluation revealed the presence of nystagmus, eccentric fixation, and poor visual acuity, with a best-corrected VA of 20/400 in both eyes. This is not different from VA obtained in early childhood, indicating that her vision loss was stationary in nature, consistent with what was volunteered in the history. Fundoscopy was largely normal, apart from decreased foveal reflex. ERG was extinguished under both photobic and scotobic conditions. The clinical profile in other affected siblings was strikingly similar (Table 1 provides detailed ophthalmological findings in all affected siblings). However, the ERG pattern of II-4 was notably different in that it was not extinguished, but rather severely decreased under photopic conditions with normal implicit time on photopic flicker, whereas scopotopic ERG was borderline, with normal oscillatory potential. Two girls (II-3 and II-4) were noticed early by the family to have strabismus, and surgical correction of the strabismus was performed in II-3. All four patients had normal intellect and growth. Other systemic examinations were unremarkable.

**Table 1:** Phenotype of patients with CABP4 mutations.

** Feature**	**This study**					**Littink et al. [****13****]**		**Zeitz et al. [****10****]**		
	**Patient 1 (II-2)**		**Patient 2 (II-3)**	**Patient 3 (II-4)**	**Patient 4 (II-6)**	**1**	**2**	**1**	**2**	**3**
Mutation	c.81_82insA					c.646C>T			c.800_801delAG	c.800_801delAG/c.370C>T
Age	16		15	12	6	12	10	39	45	15
Sex	F		F	F	M	M	F	M	M	M
Onset of vision loss	congenital		congenital	congenital	congenital	early childhood	early childhood	early childhood	early childhood	early childhood
Nystagmus	+ Resolved lately		+	+	+	+	+	+	+	-
Visual Acuity	OD	20/400	20/400	20/400	20/400	20/200	20/200	20/200	20/200	20/30
	OS	20/400	20/400	Counting Fingers	20/400	20/200	20/400	20/200	20/400	20/30
Refraction	OD	+1	+7.5	+7.5	+4.5	+5	+4.5	?	?	?
	OS	+2.5	+7.5	+7.5	+5	+5.5	+4.5	?	?	?
Fundus	Minimal changes		Normal	Normal	Normal	Minimal changes	Normal	Absent foveal reflexes	Normal	Normal
ERG	Flat		Flat	Borderline scotopic, severely decreased photopic	Flat	Normal scotopic, severely decreased photopic	Decreased scotopic, severely decreased photopic	Normal “a” wave, reduced “b” wave	Similar to but milder than (1)	Normal “a” wave, reduced “b” wave
Night blindness	No		No	No	No	No	No	No	No	Yes

Comparison of clinical and ERG characteristics of patients in this study and previously reported patients with *CABP4* mutations.

### Molecular analysis

**Figure 2:**
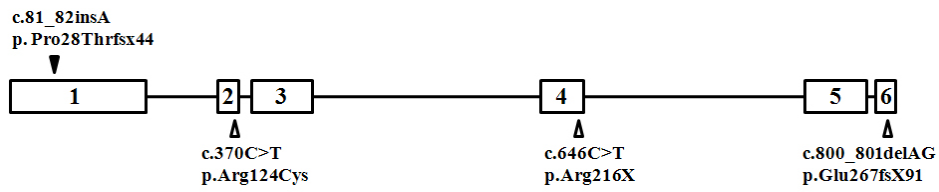
Schematic of *CABP4* gene. Previously reported mutations are indicated by empty triangles, and our novel mutation is indicated by a solid triangle.

Only one block of homozygosity was identified as shared between the four siblings. The minimal area of overlap was 3.29 Mb on chromosome 11q13, encompassing 157 genes, including *CABP4* (accession number NM_145200.2). In view of the recently described phenotypes associated with *CABP4* mutations in humans, this appeared a good candidate. Direct sequencing revealed the presence of a single base-pair insertion (c.81_82insA) in a homozygous state in all affected siblings, while parents were heterozygote carriers (Figure 1). This insertion introduced 44 novel amino acids before prematurely terminating the protein (p.Pro28Thrfsx44) (Figure 2). The transcript was, therefore, a potential target of NMD, but due to lack of availability of RNA, we were unable to test this experimentally.

## Discussion

CSNB is a rare retinal disorder characterized by functionally rather than structurally defective photoreceptors that are unable to relay their electrical impulses to the adjacent neurons. This disorder is therefore suspected when patients present with a history suggestive of retinitis pigmentosa (nyctalopia and decreased visual acuity) in the setting of a normal fundus exam. ERG is considered diagnostic, with different patterns reported in the different subtypes of CSNB [13]. Recently, Littink et al. reported a family with a novel nonsense mutation in *CABP4* and a clinical picture of decreased visual acuity, photophobia, and nystagmus, but no nyctalopia [13]. The authors of that paper noted that two of the three families reported so far with CABP4 mutations lacked nyctalopia in their clinical presentation and suggested that the term congenital cone-rod synaptic disorder be used as a more accurate name than CSNB2 for this subtype. We are in agreement with the suggestion, since this name reflects the true pathogenesis of this condition.

**Figure 3:**
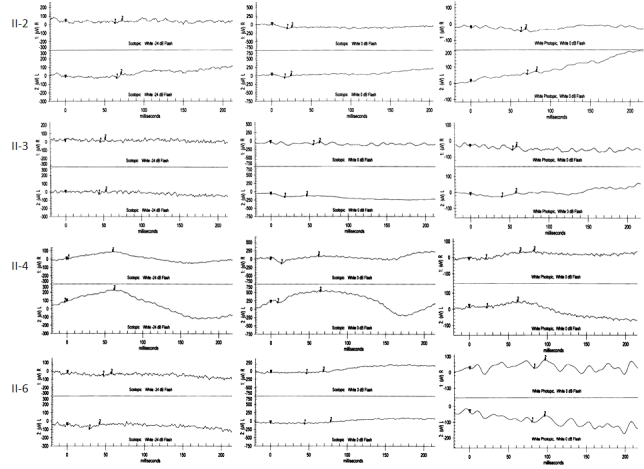
ERG findings in a family with an LCA-like phenotype secondary to the *CABP4* mutation. Scotopic and photopic ERG readings are shown for each of the four affected members, who are referred to using the IDs in Figure 1 and Table 1 (II-2, II-3, II-4, and II-6).

Table 1 summarizes the molecular and clinical characteristics of human patients with *CABP4* mutations. Three mutant alleles have been described in five patients representing three Caucasian families. Our report adds a fourth allele and four patients representing one Arab family with this disorder. Interestingly, the three previously reported mutations were most likely hypomorphic in nature. In the case of c.800_801delAG, it was predicted to elongate the otherwise 276 amino acid-long CABP4 by another 91 novel amino acids (p.Glu267fsX91). The introduction of the novel amino acids was downstream of the last Ca-binding motif, making it unlikely to directly interfere with that function of the protein [10]. However, this mutation was found to significantly reduce the abundance of the wild-type transcript, so the authors speculated that a combination of change in the tertiary structure and a reduction in transcriptional efficiency was responsible for the pathogenicity of this mutation [10]. Interestingly, this mutation was found to display a founder effect in another patient who was compound heterozygous for the only missense mutation reported in *CABP4* (Arg124Cys) [10]. It is unclear how this missense mutation adversely affects the protein function, since it does not reside within any of the four Ca-binding motifs. Indeed, the authors speculated the effect may in fact be at the level of transcription, because compound heterozygosity for this mutation and c.800_801delAG, just like homozygosity for the latter, was associated with decreased abundance of the wild-type transcript [10]. The third mutation, p.Arg216X, was also speculated to be hypomorphic in nature, since it does not undergo NMD and only truncates two of the four Ca-binding motifs [13]. Our mutation, on the other hand, is very likely to be a complete null. Even if the mutant transcript escapes NMD, the introduction of novel amino acids very early in the protein will abolish all Ca-binding motifs and essentially render the protein functionally absent (Figure 2). Therefore, our mutation is predicted to result in the most dramatic functional perturbation of this important protein, and this may explain the more severe phenotype observed in our patients, compared to others (Table 1). LCA and CSNB are usually considered distinct clinical entities. The congenital onset of symptoms in our patients and their extinguished ERG (Figure 3) argue for their classification as LCA [16]. However, lack of enophthalmos and Franceschetti's oculo-digital sign in our patients must be noted. The normal appearance of the fundus in the four patients should not be viewed as evidence against LCA, since GUCY2D-related LCA is known to be associated with a normal-looking fundus, even in adults [17]. However, the stationary nature of the disease observed in our four patients does complicate their classification as LCA, which is known to be progressive in nature. Nonetheless, this family provides a unique opportunity to observe a severe retinal phenotype that resembles LCA caused by a mutation in a gene hitherto described as a CSNB gene.

In summary, our study is in line with our previous reports of the clinical utility of homozygosity mapping in the setting of genetically heterogeneous disorders. It expands on the retinal phenotype associated with mutations in *CABP4*, and makes it necessary to consider this gene in the context of LCA-like presentations with extinguished ERG.
